# Effect and maintenance of the SLIMMER diabetes prevention lifestyle intervention in Dutch primary healthcare: a randomised controlled trial

**DOI:** 10.1038/nutd.2017.21

**Published:** 2017-05-08

**Authors:** G Duijzer, A Haveman-Nies, S C Jansen, J ter Beek, R van Bruggen, M G J Willink, G J Hiddink, E J M Feskens

**Affiliations:** 1 Division of Human Nutrition, Wageningen University, Academic Collaborative Centre AGORA, Wageningen, The Netherlands; 2 GGD Noord- en Oost-Gelderland (Community Health Service), Warnsveld, The Netherlands; 3 Huisartsenzorg Regio Apeldoorn, Apeldoorn, The Netherlands; 4 BV Diabeteszorg Oude IJssel, Doetinchem, The Netherlands; 5 Wageningen University, Strategic Communication, Sub-department Communication, Philosophy and Technology, Centre for Integrative Development, Social Sciences, Wageningen, The Netherlands

## Abstract

**Background/Objectives:**

To assess the effectiveness of the SLIMMER combined dietary and physical activity lifestyle intervention on clinical and metabolic risk factors, dietary intake, physical activity, and quality of life after 12 months, and to investigate whether effects sustained six months after the active intervention period ended.

**Subjects/Methods:**

SLIMMER was a randomised controlled intervention, implemented in Dutch primary healthcare. In total, 316 subjects aged 40–70 years with increased risk of type 2 diabetes were randomly allocated to the intervention group (10-month dietary and physical activity programme) or the control group (usual healthcare). All subjects underwent an oral glucose tolerance test and physical examination, and filled in questionnaires. Identical examinations were performed at baseline and after 12 and 18 months. Primary outcome was fasting insulin.

**Results:**

The intervention group showed significantly greater improvements in anthropometry and glucose metabolism. After 12 and 18 months, differences between intervention and control group were -2.7 kg (95% confidence interval (CI): −3.7; −1.7) and −2.5 kg (95% CI: −3.6; −1.4) for weight, and −12.1 pmol l^−1^ (95% CI: −19.6; −4.6) and −8.0 pmol l^−1^ (95% CI: −14.7; −0.53) for fasting insulin. Furthermore, dietary intake, physical activity, and quality of life improved significantly more in the intervention group than in the control group.

**Conclusions:**

The Dutch SLIMMER lifestyle intervention is effective in the short and long term in improving clinical and metabolic risk factors, dietary intake, physical activity, and quality of life in subjects at high risk of diabetes.

## Introduction

Universal consensus exists on the need to translate and implement evidence from landmark clinical trials on combined lifestyle interventions to prevent type 2 diabetes in real-world settings.^1^ Recent reviews on studies conducted in such settings showed limited results, with significant reductions in weight and waist circumference but inconclusive findings for metabolic indicators of diabetes risk, such as blood glucose or HbA1c.^2, 3, 4^ Furthermore, current evidence on sustainability and long-term clinical benefits of such interventions is limited.^2, 3, 4^ To date, no evidence-based diabetes prevention interventions have been effectively implemented in Dutch primary healthcare,^5, 6^ while the Study on Lifestyle intervention and Impaired glucose tolerance Maastricht (SLIM), conducted in an experimental setting, had earlier revealed a 47% diabetes risk reduction.^7^ We therefore used the SLIM intervention as a starting point for implementation and translated this into the SLIMMER intervention (SLIM iMplementation Experience Region Noord- en Oost-Gelderland). This translation was done jointly by SLIM intervention developers and local healthcare professionals.^8^ Pilot-testing of the adapted intervention showed its implementation was feasible in Dutch primary healthcare and that it was likely to achieve desired impact.^9^ These results served as input for broader implementation and evaluation of the intervention.^10^ In this study, we assess the effectiveness of the SLIMMER intervention on clinical and metabolic risk factors, dietary intake, physical activity (PA), and quality of life after 12 months. Moreover, the aim is to investigate whether effects sustained six months after the active intervention period ended.

## Materials and methods

### Study design

The SLIMMER study’s design and 10-month lifestyle intervention programme have been described in detail elsewhere.^10^ In short, SLIMMER was a randomised, controlled intervention study, conducted in the cities of Apeldoorn and Doetinchem (the Netherlands). It was implemented in Dutch public health and primary healthcare, involving 25 general practices—general practitioners (GPs) and their practice nurses—, 11 dieticians, 16 physiotherapists and 15 sports clubs. The study protocol was approved by the Wageningen University Medical Ethics Committee, and all subjects gave their written informed consent before the study started. The SLIMMER study is registered with ClinicalTrials.gov (Identifier NCT02094911).

### Study population

Study subjects were recruited by GPs and practice nurses from their patient registration database, using either a laboratory glucose test or the Dutch Diabetes Risk Test. Inclusion criteria were (1) aged between 40 and 70 years at screening, (2) impaired fasting glucose (IFG; 6.1–6.9 mmol l^−1^) or an elevated/high risk of type 2 diabetes (a Diabetes Risk Test score of ⩾7 points), (3) willing and able to participate in the study for at least 1.5 years, and (4) able to speak and understand the Dutch language. Exclusion criteria were, amongst others, known diabetes and any severe cardiovascular or psychiatric disease. Criteria were checked using electronic medical records. There were no racial or gender criteria. Recruitment took place between October 2011 and September 2012 in three consecutive groups for logistical reasons.

**Figure 1:**
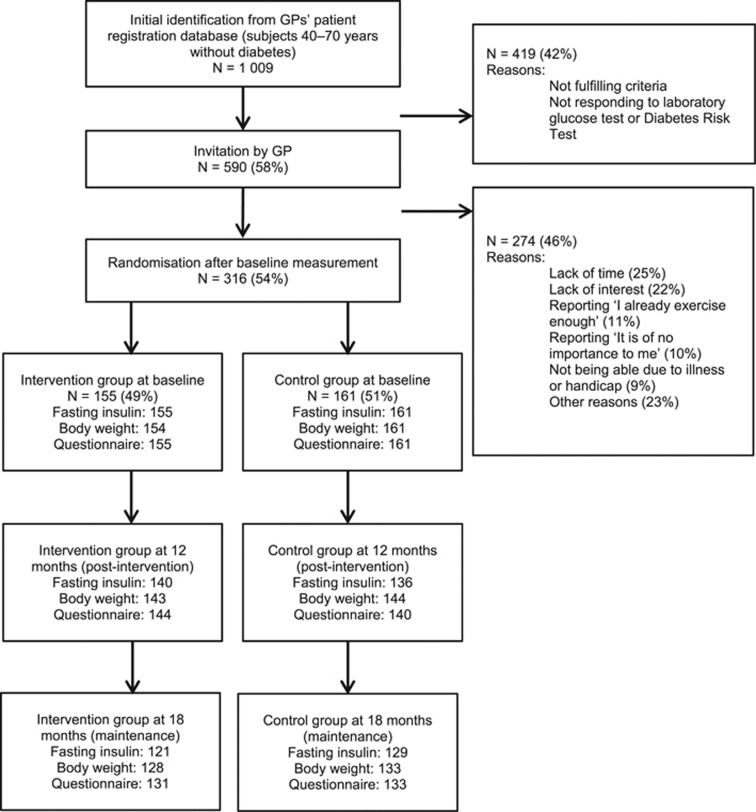
Flow diagram.

In total, 1009 individuals aged 40–70 years without diabetes mellitus were initially identified from the patient registration database (Figure 1). Of these, 590 (58%) fulfilled all criteria and were invited to participate. In total, 316 subjects (54%) were willing to participate and underwent an oral glucose tolerance test (OGTT) and physical examination at baseline.

After baseline measurements, participants were randomly allocated to the intervention or control group (allocation ratio 1:1), using block randomisation at GP level (permuted blocks with size 2) and stratification for sex. Couples were allocated to the same group to avoid contamination. An independent research assistant from the Division of Human Nutrition, Wageningen University, performed the randomisation, using a computerised random number generator. Allocation concealment was ensured, as allocation was not announced until baseline measurements were completed. One of the researchers (GD) informed participants on the assignment to the intervention or control group.

### Intervention

The SLIMMER combined lifestyle intervention resembled the SLIM intervention,^7^ which was based on the Finnish Diabetes Prevention Study,^11^ and consisted of a dietary and a PA component, delivered by primary healthcare professionals (GPs, practice nurses, dieticians and physiotherapists).^10^ Furthermore, case management and a maintenance programme were included. The dietary intervention consisted of tailored dietary advice given by a dietician, during five to eight individual consultations (average of 5.6 consultations per participant) and one group session. The aim was to adopt, step by step, a sustainable, healthy dietary pattern according to the Dutch dietary guidelines. Furthermore, the intervention aimed to help overweight participants to achieve 5–10% weight loss. The PA intervention was delivered by physiotherapists as weekly group-based combined aerobic and resistance training sessions (average of 38 sports lessons per participant), based on the Dutch guidelines for PA and type 2 diabetes.^12^ The aim was to obtain and maintain an active lifestyle, which includes moderate-intensity PA for at least 30 min per day on at least five days a week. Furthermore, case management was performed by practice nurses (contacting intervention participants and healthcare professionals by phone) to enhance participant compliance and feasibility of implementation.

In addition to the core dietary and PA intervention, a maintenance programme was delivered, starting in the last phase of the 10-month intervention period and lasting up to three months thereafter. This programme comprised sports clinics at local sports clubs, concluding meetings with the dietician and physiotherapist, and a return session with the physiotherapist, dietician, and the PA group.^13^ This programme was added to guide participants in the process of maintaining lifestyle behaviour change in an independent and sustainable manner.

Control group subjects received usual healthcare as provided by GPs and practice nurses (yearly monitoring of blood glucose, according to the guidelines of the Dutch College of General Practices).^14^ Furthermore, at baseline they received written information on the beneficial effects of a healthy diet and increased PA. No additional appointments were scheduled, apart from visits for follow-up measurements.

### Data collection and outcome measures

Baseline measurements were taken between February and October 2012. All study subjects underwent an OGTT and physical examination, and filled in questionnaires. Identical examinations were performed at baseline, after 12 months (at the end of the intervention; between February and September 2013), and after 18 months (6 months after the end of the intervention; between September 2013 and March 2014; Supplementary Figure S1). These procedures have previously been described in detail.^10^

The primary outcome was fasting insulin, determined on the basis of a standard OGTT with a glucose load of 75 g, performed by trained nurses after at least 10 h of fasting. Fasting and 2-h plasma glucose levels, HbA1c, and serum lipids (cholesterol (total, HDL and LDL) and triglycerides) were determined at SHO laboratory in Velp, The Netherlands. For fasting insulin, all blood samples were analysed within one run after 18 months. An index for insulin resistance was calculated from fasting plasma glucose and insulin concentration, using the homoeostasis model assessment (HOMA-IR). Diabetes was classified based on World Health Organization recommendations^15, 16^ and standards of the American Diabetes Association.^17^ Normoglycaemia was defined as fasting glucose <6.1 mmol l^−1^ and 2-h glucose <7.8 mmol l^−1^; isolated IFG was defined as fasting glucose 6.1–6.9 mmol l^−1^ and 2-h glucose <7.8 mmol l^−1^; impaired glucose tolerance was defined as fasting glucose <7.0 mmol l^−1^ and 2-h glucose 7.8–11.0 mmol l^−1^; and diabetic values were defined as fasting glucose ⩾7.0 mmol l^−1^, 2-h glucose ⩾11.1 mmol l^−1^, HbA1c ⩾6.5%, or using diabetes medication. Clinical assessments were performed by trained research assistants in research centres in Apeldoorn and Doetinchem according to standardised procedures. BMI was calculated as the ratio of weight and height squared (kg m^−^^2^). Waist circumference was obtained at the level midway between the lowest rib and the iliac crest. Blood pressure was measured using the Omron Digital Blood Pressure Monitor HEM-907.

Socio-demographic characteristics (age, sex, education level, ethnic background, smoking and family history of diabetes) were collected by participant questionnaires. These data were collected according to standards of the national surveillance system in the Netherlands and an existing questionnaire. Self-reported medication use was determined using a questionnaire. Non-response data (age, sex, reason for non-participation, perceived health, and education level) were collected during the recruitment period by practice nurses.

Dietary intake (nutrient intake and food intake) was assessed by a validated Food Frequency Questionnaire (FFQ). FFQs were checked by trained research assistants. Average daily nutrient intakes were calculated by multiplying frequency of consumption by portion size and nutrient content per gram using the 2011 Dutch food composition table.^18^ Food intake behaviours were formulated based on Dutch food-based dietary guidelines and common dietician practices in the SLIMMER pilot study.^9^ Adherence to the Dutch dietary guidelines was calculated, based on the Dutch Healthy Diet Index (DHD-index).^19^ The original DHD-index consisted of 10 components representing the guidelines, whereas for the current study we adapted the index and included eight components: PA, vegetable, fruit, fibre, fish (EPA and DHA), saturated fat, trans-fatty acids, and alcohol. Two components (‘acidic drinks and foods’ and ‘sodium’) were excluded because no data were available on these components. Per component, the score ranged between 0 and 10, resulting in a total score between 0 (no adherence) and 80 (complete adherence).

PA was measured using the validated Short QUestionnaire to Assess Health-enhancing physical activity (SQUASH). The durations (minutes per week) of total and light-, moderate-, and vigorous-intensity physical activities were calculated. Level of compliance with the PA guidelines (moderate-intensity PA for at least 30 min per day on at least five days a week) was represented as inactive (0 days), semi-active (1–4 days), or norm-active (at least 5 days).^20^ Furthermore, physical fitness was measured as the distance covered in metres during the six-minute walk test.

Quality of life was assessed by the Short-Form Health Survey (SF-36), which has proved to be a practical, reliable, and valid tool for both general and chronic disease populations in the Netherlands. The questions were organised into one item on health transition and eight scales for, respectively, physical functioning, role limitations due to physical health problems, bodily pain, general health perceptions, vitality, social functioning, role limitations due to emotional problems, and general mental health. The eight scales were converted to a 0–100 scale indicating worst to best possible health. Scores were summarised into the physical component summary score and the mental component summary score.

### Statistical analysis

A sample size of 145 subjects per group was required to detect differences between groups in fasting insulin, assuming an alpha of 0.05, power of 80%, two-sided test, and an expected drop-out rate of 10%. After exclusion of subjects because of missing data on fasting insulin or BMI at baseline or 12 months (*n*=16 in the intervention group and *n*=25 in the control group), data collected from 275 subjects were used for statistical analysis. Participants who dropped out were not substantially different from the completers in baseline characteristics, except that drop-outs were more often divorced than completers (25 vs 6% in the intervention group and 28 vs 5% in the control group). Furthermore, the HOMA-IR was higher in intervention drop-outs than in completers (3.1±2.8 vs 2.0±1.1, *P*=0.053), whereas this was lower in control drop-outs than in completers (1.5±0.6 vs 2.0±1.2, *P*=0.015).

Continuous variables are presented as mean±s.d. and categorical variables as percentages. Natural log transformations were used in the event of skewed distributions. Differences within groups were tested for statistical significance with paired samples *t*-tests for normally distributed variables and Wilcoxon signed-rank tests for non-normally distributed variables. Differences between groups were tested for statistical significance with independent samples *t*-tests for normally distributed variables and Mann–Whitney tests for non-normally distributed variables, *χ*^2^-tests and analysis of covariance adjusting for baseline value, sex, recruitment phase, and medication use if applicable. Additional analyses showed similar results when subjects on medication were excluded. We included an interaction term in the analysis of covariance models to test whether the association between treatment and outcome measures differed by sex. All primary analyses were performed according to the intention-to-treat principle: participants were analysed in the groups to which they were originally randomly assigned, regardless of whether or not they actually participated in the intervention. All analyses were performed using IBM SPSS Statistics version 22 (IBM Corp., Armonk, NY, USA).

## Results

### Baseline results

Study subjects and non-responders (those who were not willing to participate) were similar in terms of sex, age and education level. However, 90% of the non-responders perceived their health as good or even better, against 79% of the study subjects. The most important reasons for non-response were lack of time (25%), lack of interest (22%), reporting ‘I already exercise enough’ (11%), reporting ‘It is of no importance to me’ (10%), and not being able due to illness or handicap (9%).

Table 1 shows the baseline characteristics of the 275 subjects. No differences in baseline characteristics between study groups were found. On average, subjects were 61 years old, and most had a low education level, were Dutch, and had a family history of diabetes. Of the total, 48% were overweight (BMI ⩾25 and <30 kg m^−2^), 42% were obese (BMI⩾30 kg m^−2^), and 15% had a cardiovascular disease in the past (data not shown). Dietary intake was similar between groups both in terms of nutrient intake and food intake, and both groups had similar adherence to the Dutch dietary guidelines. Moderate-to-vigorous intensity PA was comparable between groups and 80% of participants were classified as norm-active. Health-related quality of life was comparable in both groups, with scores around 50 for both physical and mental component scores.

### Results on clinical and metabolic risk factors

Table 2 summarises changes in clinical and metabolic risk factors after 12 and 18 months. Beneficial changes were observed in the intervention group compared with the control group. At 12 months, mean weight reduction was 3.4% in the intervention group and 0.3% in the control group (*P*<0.001). Furthermore, waist circumference reduction was greater in the intervention group. Fasting insulin declined more in the intervention group than in the control group (−12.6 vs 0.6 pmol l^−1^, *P*=0.005). Also, greater improvements were seen in fasting glucose (−0.2 vs −0.01 mmol l^−1^), 2- h glucose (−0.5 vs 0.2 mmol l^−1^), HbA1c (−0.15 vs −0.07%), and HOMA-IR (−0.29 vs 0.02) in the intervention group than in the control group (*P*<0.05). Compared to baseline, more subjects had normoglycaemia (19 vs 25% in the intervention group and 15 vs 20% in the control group) and fewer intervention subjects (32 vs 27%) than control subjects (28 vs 31%) had diabetic values. No significant differences in serum lipids between groups were observed. Systolic and diastolic blood pressure reduced in both groups. However, no significant differences between groups were noted.

No differences in outcomes were observed between subjects recruited by laboratory glucose test (*n*=130) or by Diabetes Risk Test (*n*=110), except for fasting glucose at 12 months: in subjects recruited by laboratory glucose test, fasting glucose was significantly lower in the intervention group than in the control group (*β*=−0.4, 95% confidence interval (CI) −0.6; −0.1), whereas there was no effect on fasting glucose in subjects recruited by diabetes risk test (*β*=−0.0, 95% CI −0.2; 0.2; *P* for interaction=0.016; Supplementary Table S1).

At 18 months, reductions in weight, waist circumference, fasting glucose, 2-h glucose, fasting insulin, HbA1c, and HOMA-IR were sustained in favour of the intervention group (Table 2). Even more subjects than at 12 months had normoglycaemia (44% in the intervention group and 38% in the control group).

### Results on dietary intake and PA

At 12 months, intake of energy, fat, and saturated fat was reduced; intake of fibre increased more in the intervention group than in the control group (Table 3). No significant differences in intake of protein, carbohydrates and alcohol between groups were observed. Fruit intake increased significantly in the intervention group but decreased in the control group. No significant difference in vegetable intake between groups was found. Generally, similar results were observed when food groups were expressed per 1000 kcal. The DHD-index score improved significantly more in the intervention group than in the control group (3.6 vs 0.3, *P*<0.001, Table 3).

At 18 months, no effect on energy and protein intake was found, whereas intake of fat and saturated fat reduced even more than at 12 months in the intervention group compared with the control group (Table 3), especially in men (data not shown). Furthermore, the effect on fibre intake continued at 18 months. No significant difference in fruit intake between groups was observed anymore, in contrast to vegetable intake, which was significantly higher in the intervention group than in the control group (15.7 vs 2.2 g, *P*=0.039). Also at 18 months, the DHD-index score was significantly higher in the intervention group than in the control group.

At 12 months, the intervention group spent more time on vigorous activities compared with baseline, whereas this decreased in the control group (65.7 vs −80.2 min per week, *P*=0.006; Table 3). Furthermore, the intervention group improved more on physical fitness than the control group (covered distance 25.1 vs 2.3 m, *P*<0.001).

At 18 months, the intervention group further increased time spent on vigorous activities. However, the control group also slightly improved (Table 3). Especially women in the intervention group spent more time on vigorous activities than women in the control group (at 12 months *p* for interaction=0.055, and at 18 months *p* for interaction=0.051). Furthermore, the improvement in physical fitness was maintained in favour of the intervention group (Table 3).

### Results on quality of life

At 12 months, the item ‘health transition’ and the sub-scales ‘physical functioning’ and ‘general mental health’ improved in the intervention group compared with the control group (*P*<0.05; Table 4). Additional analyses showed that the sub-scale ‘role limitations due to physical health problems’ improved in women in the intervention group but not in men (*P* for interaction=0.014). No significant differences in physical component score or mental component score between groups were observed (Table 4).

At 18 months, the effect on health transition continued and, additionally, significant effects were found for ‘general health perceptions’, ‘role limitations due to emotional problems’, and ‘social functioning’ in favour of the intervention group (Table 4). Moreover, the mental component score significantly improved in the intervention group compared with the control group (2.4 vs −0.1), but no effect was found on the physical component score.

## Discussion

The aim of this study was to assess the effectiveness and maintenance of the SLIMMER lifestyle intervention after 12 and 18 months in Dutch primary healthcare. It was shown that the SLIMMER intervention improved body weight, clinical and metabolic risk factors, dietary intake, physical activity, and quality of life. Furthermore, it was shown that most of these improvements sustained at 18 months.

It is often shown that the effectiveness of lifestyle interventions in real-world settings is limited compared with experimental settings,^3^ due to the real world’s complexity and limited finance and resources.^21^ The SLIMMER lifestyle intervention, however, showed a weight reduction of 3.0 kg after 12 months, which is comparable with that in the original SLIM study (−2.8 kg).^7^ Furthermore, our study found better improvements in several clinical and metabolic risk factors, such as weight, BMI, waist circumference, fasting and 2-h glucose, and HbA1c, compared with most other real-world programmes.^3, 4, 5^ These results were found in two primary healthcare settings that are representative of Dutch primary healthcare.

Weight reduction during our study can be indicated as modest. However, it is still relevant, as several studies have shown that even modest weight reduction can reduce the risk of diabetes.^22, 23^ In the US Diabetes Prevention Program, it was shown that diabetes incidence can be reduced by around 16% for each kilogram of weight lost.^22^ Given the weight loss seen in the SLIMMER intervention group compared with the control group, we would expect around a 43% reduction in type 2 diabetes attributable to weight loss at 12 months, and a 40% reduction at 18 months, which is comparable with the 47% risk reduction in the SLIM study.^7^ A review of 36 studies assessing diabetes prevention in real-world settings revealed a 26% risk reduction, which is lower than our results, possibly because of the less intensive nature of many included interventions.^4^

Weight reduction is maintained at 18 months. This is remarkable, as it is well known that weight regain following the end of an intensive lifestyle programme is common within five years,^24^ even in successful lifestyle interventions.^25, 26^ This result might partly be explained by the inclusion of a maintenance programme following the intensive lifestyle programme. It is suggested that such a maintenance programme could enhance intervention effectiveness because of the use of specific behaviour change techniques such as goal-setting, self-monitoring, and relapse prevention.^27^ However, data on weight loss maintenance in real-world trials is limited, as very few studies report outcomes beyond 12 months.^4^ Our study investigated maintenance after six months. However, benefits over extended follow-up should be further investigated.

Our results indicate that the SLIMMER intervention was successful in improving overall dietary patterns and several nutrient intakes, such as total fat, saturated fat, and fibre intake. Furthermore, vigorous physical activities were more improved in the intervention group than in the control group. The Dutch Aphrodite lifestyle intervention found beneficial effects only for total physical activities and fibre intake.^5^ A systematic review of diabetes prevention interventions in the real world, however, concluded that, overall, changes in diet and PA are poorly reported, and that more research is needed.^4^

Several factors in our lifestyle programme compared to others could have contributed to intervention effectiveness. First, the SLIMMER intervention was highly intensive, with weekly sports lessons and regular dietary consultations for 10 months. Several reviews found that increased intervention effectiveness was associated with higher intervention intensity.^3, 4, 27^ Second, lifestyle advice in our study was provided by dieticians and physiotherapists rather than by more general lifestyle coaches in other studies, such as GPs or practice nurses (or both) and lay community educators.^27^ Specialist professionals are more specifically educated for, and more experienced in, delivering nutritional or physical activity advice, and this may have contributed to intervention effectiveness. Moreover, several international reviews concluded that a wide range of staff can deliver effective interventions.^4, 27^ Third, the thorough preparation of the SLIMMER intervention may have contributed to its effectiveness.^28^ Much attention was paid to carefully translating the intervention programme to the real world in a joint decision-making process with intervention developers and local healthcare professionals,^8^ followed by pilot-testing of the adapted intervention programme^9^ prior to implementation and evaluation.

Risk scores might be good tools to screen people at high risk of type 2 diabetes in primary healthcare. They are non-invasive, easy, and cheap compared with fasting plasma glucose measurements. The Diabetes Risk Test used in the current study is based on the FINDRISC questionnaire, which has been shown to be capable of predicting undiagnosed diabetes and prediabetes.^29^ As shown in our study, intervention subjects improved weight and glucose tolerance, independent of manner of recruitment (fasting plasma glucose or Diabetes Risk Test). This is in line with the review by Ashra *et al.*^4^

### Strengths and limitations

The randomised design, comprehensive evaluation approach (outcomes at several levels), and validated methods to measure dietary intake and PA allow us to draw solid conclusions on the SLIMMER intervention’s effectiveness. By investigating outcomes at several levels (dietary intake and PA alongside clinical and metabolic risk factors), we now have more insight into determinants contributing to diabetes prevention, such as intakes of fat, saturated fat and fibre, and vigorous activities. However, it should be noted that dietary intake and physical activity were based on self-reported data. Although we observed beneficial changes in quality of life, many were non-significant. Therefore, a disease-specific questionnaire might have been used rather than the SF-36 questionnaire, as such a generic instrument is less responsive to changes in health-related quality of life.^30^

## Conclusions

In summary, this study has shown that the Dutch SLIMMER lifestyle intervention is effective in the short and long term in improving clinical and metabolic risk factors, dietary intake, physical activity, and quality of life in subjects at high risk of diabetes. More insight into longer-term effects of the intervention on maintenance and cost-effectiveness is needed and important for sustainable diabetes prevention. The results provide valuable information for primary healthcare professionals, researchers and policymakers.

## Figures and Tables

**Table 1:** Baseline characteristics of participants in the SLIMMER intervention (*n*=275)^a^

	*INT (*n=*139)*	*CON (*n=*136)*
*Socio-demographics*		
*Sex (n, %)*		
Male	75 (54)	69 (51)
Female	64 (46)	67 (49)
Age (years)	61.1±6.1	61.2±6.6
		
*Education level (n, %)*^b^		
Low	74 (53)	73 (54)
Middle	39 (28)	26 (19)
High	26 (19)	37 (27)
		
*Ethnicity (n, %)*		
Dutch	123 (88)	123 (90)
Western non-Dutch	12 (9)	9 (7)
Non-western non-Dutch	4 (3)	4 (3)
		
*Family history of diabetes (n, %)*		
No	46 (33)	57 (42)
First degree	66 (48)	62 (46)
Second degree	27 (19)	17 (12)
		
*Smoking status (n, %)*		
Yes	21 (15)	26 (19)
Ex-smoker	86 (62)	78 (57)
No, never	32 (23)	32 (24)
		
*Clinical and metabolic risk factors*		
Weight (kg)	89.5±17.0	87.8±15.2
BMI (kg/m^2^)	30.2±4.5	29.9±4.8
		
*Waist circumference (cm)*		
Male	109.3±12.2	107.4±10.2
Female	101.0±12.0	100.0±12.9
Fasting glucose (mmol l^−1^)	6.6±0.6	6.6±0.6
2-h glucose (mmol l^−1^)^c^	8.2±2.8	8.0±2.5
Fasting insulin (pmol l^−1^)	87.8±48.2	86.0±52.8
HOMA-IR	2.0±1.1	2.0±1.2
HbA1c (% (mmol mol^−1^))	5.8±0.4 (40.3±3.9)	5.8±0.4 (40.1±4.0)
Total cholesterol (mmol l^−1^)	5.5±1.1	5.5±1.2
HDL cholesterol (mmol l^−1^)	1.5±0.4	1.4±0.4
LDL cholesterol (mmol l^−1^)	3.5±1.0	3.6±1.0
Triglycerides (mmol l^−1^)	1.7±0.9	1.6±0.8
		
*Blood pressure (mm Hg)*^c^		
Systolic	133.0±15.5	131.0±13.5
Diastolic	77.5±10.7	75.6±8.7
		
*Dietary intake*		
Energy intake (kcal per day)	1986.3±576.1	2040.7±634.2
Saturated fat (en%)	11.8±2.2	11.9±2.3
Fibre (g per 1000 kcal)	11.0±2.6	11.1±2.4
Alcohol (en%)	4.8±5.7	4.3±5.2
Fruit intake incl. fruit juices (g per day)	186.1 ±119.9	206.5±140.0
Vegetable intake (g per day)	149.3±96.6	137.8±84.7
Fibre intake from total bread intake (%)	5.6±1.0	5.8±1.2
Fat intake from total bread spread intake (%)	21.0±6.4	19.6±6.1
Snack intake (kcal per day)	278.5±208.2	323.0±272.7
Soft drink intake (kcal per day)	56.3±69.6	48.5±60.1
Dutch Healthy Diet index (0–80 scale)	58.8±9.5	58.7±9.0
		
		
*Physical activity (PA)*		
Total PA (min per week)	2254±1337	2306±1232
Light PA (min per week)	1307±1094	1331±970
Moderate PA (min per week)	593±692	559±552
Vigorous PA (min per week)	354±427	417±450
Physical fitness (m)^c^	454.0±58.0	455.5±57.7
		
*Quality of life*		
Physical component score	50.1±8.2	49.8±7.9
Mental component score	50.1±10.3	50.7±8.1

Abbreviations: BMI, body mass index; CON, Control group; HbA1c, glycated haemoglobin; HDL, high-density lipoprotein; HOMA-IR, homoeostasis model assessment insulin resistance; INT, Intervention group; LDL, low-density lipoprotein; PA, physical activity.

a Data are *n* (%) or mean±s.d.

b Education level was based on the highest level of education completed and divided in three categories: low (no, primary or lower secondary school), middle (higher secondary school or intermediate vocational school), and high (higher professional education or university level).

c 2- h glucose INT *n*=138, CON *n*=136; systolic blood pressure INT *n*=138, CON *n*=135; diastolic blood pressure INT *n*=138, CON *n*=135; physical fitness INT *n*=138, CON *n*=136.

**Table 2:** Changes in clinical and metabolic risk factors from baseline to 12 months (*n*=275) and 18 months (*n*=240)^a^

	*From baseline to 12 months*			*From baseline to 18 months*		
	*INT*	*CON*	β *(95% CI)*^b^	*INT*	*CON*	β *(95% CI)*^b^
*n*	139	136		118	122	
Weight (kg)	−3.0±5.1^c^	−0.3±3.6	−2.7 (−3.7; −1.7)	−2.9±5.1^c^	−0.4±3.7	−2.5 (−3.6; −1.4)
BMI (kg m^−2^)	−1.0±1.7^c^	−0.1±1.2	−0.9 (−1.3; −0.6)	−1.0±1.7^c^	−0.1±1.3	−0.8 (−1.2; −0.5)
						
*Waist circumference (cm)*						
Men	−5.4±5.1^c^	−2.0±4.8^c^	−3.2 (−4.8; −1.6)	−4.9±5.6^c^	−2.4±5.7^c^	−2.3 (−4.3; −0.3)
Women	−5.2±6.0^c^	−0.8±4.2	−4.4 (−6.1; −2.6)	−3.8±6.7^c^	−0.2±4.9	−3.4 (−5.5; −1.3)
Fasting glucose (mmol l^−1^)	−0.2±0.7^c^	−0.0±0.7	−0.2 (−0.3; −0.0)	−0.6±0.6^c^	−0.4±0.6^c^	−0.2 (−0.3; −0.0)
2-h glucose (mmol l^−1^)^c^	−0.5±2.2^c^	0.2±2.6	−0.8 (−1.3; −0.3)	−1.0±2.1^c^	−0.3±2.5^c^	−0.7 (−1.2; −0.1)
Fasting insulin (pmol l^−1^)	−12.6±36.9^c^	0.6±38.1	−12.1 (−19.6; −4.6)	−17.9±35.5^c^	−9.3±38.5^c^	−8.0 (−14.7; −0.53)
HOMA-IR	−0.29±0.85^c^	0.02±0.86	−0.28 (−0.46; −0.11)	−0.45±0.81^c^	−0.25±0.85^c^	−0.19 (−0.34; −0.02)
HbA1c (% (mmol mol^−1^))	−0.15±0.21^c^	−0.07±0.23^c^	−0.09 (−0.14; −0.04)	−0.17±0.21^c^	−0.06±0.45^c^	−0.11 (−0.20; −0.02)
	−1.67±2.29^c^	−0.74±2.49^c^	−0.99 (−1.55; −0.42)	−1.86±2.31^c^	−0.62±4.95^c^	−1.18 (−2.14; −0.21)
Total cholesterol (mmol l^−1^)	−0.07±0.88	−0.10±0.96	0.02 (−0.16; 0.20)	−0.17±0.83^c^	−0.16±1.00	−0.04 (−0.24; 0.16)
HDL cholesterol (mmol l^−1^)	0.02±0.18	0.01±0.15	0.01 (−0.03; 0.05)	0.01±0.21	0.04±0.18^c^	−0.02 (−0.07; 0.02)
LDL cholesterol (mmol l^−1^)	−0.05±0.82	−0.14±0.88	0.05 (−0.11; 0.21)	−0.05±0.78	−0.06±0.95	−0.06 (−0.25; 0.14)
Triglycerides (mmol l^−1^)	−0.08±0.67	0.03±0.73	−0.09 (−0.25; 0.07)	−0.01±0.63	0.03±0.57	−0.02 (−0.16; 0.13)
Systolic blood pressure (mm Hg)^d^	−2.8±11.4^c^	−1.8±11.4	−0.3 (−2.8; 2.2)	−1.9±12.9	−2.7±11.6^c^	1.0 (−2.0; 3.9)
Diastolic blood pressure (mm Hg)^d^	−4.0±7.4^c^	−2.4±7.0^c^	−1.0 (−2.6; 0.6)	−2.6±8.4^c^	−2.7±6.9^c^	0.1 (−1.7; 1.9)

Abbreviations: BMI, body mass index; CI, confidence interval; CON, Control group; HbA1c, glycated haemoglobin; HDL, high-density lipoprotein; HOMA-IR, homoeostasis model assessment insulin resistance; INT, Intervention group; LDL, low-density lipoprotein; PA, physical activity.

a Data are mean±s.d. or β (95% CI).

b *β* (95% CI) for fasting glucose, 2-h glucose, fasting insulin, HOMA-IR, HbA1c, total cholesterol, HDL cholesterol, LDL cholesterol, triglycerides, systolic and diastolic blood pressure were adjusted for medication use.

c Significant difference within group (*P*<0.05).

d From baseline to 12 months: 2-h glucose: INT *n*=134, CON *n*=132; systolic and diastolic blood pressure: INT *n*=136, CON *n*=130; From baseline to 18 months: 2-h glucose: INT *n*=113, CON *n*=118; systolic and diastolic blood pressure: INT *n*=113, CON *n*=119.

**Table 3:** Changes in dietary intake and physical activity from baseline to 12 months (*n*=272) and 18 months (*n*=239)^a^

	*From baseline to 12 months*^b^			*From baseline to 18 months*^b^		
	*INT*	*CON*	β *(95% CI)*	*INT*	*CON*	β *(95% CI)*
*n*	139	134		117	122	
						
*Dietary intake*						
Energy intake (kcal per day)	−233.5±452.5^c^	−178.8±538.7^c^	−93.2 (−186.3;−0.2)	−226.3±453.7^c^	−255.3±518.8^c^	−5.2 (−104.3; 93.9)
Total protein (en%)	0.7±2.6^c^	0.3±2.29	0.4 (−0.2; 0.9)	0.8±2.7^c^	0.7±2.6^c^	0.2 (−0.4; 0.8)
Total fat (en%)	−1.5±5.1^c^	−0.4±4.5	−1.2 (−2.2; −0.1)	−1.6±5.4^c^	−0.1±5.1	−1.6 (−2.8; −0.4)
Saturated fat (en%)	−0.9±2.3^c^	−0.2±2.0	−0.7 (−1.2; −0.3)	−1.0±2.3^c^	−0.1±1.9	−0.9 (−1.4; −0.4)
Total carbohydrates (en%)	1.0±5.1^c^	0.3±4.7	0.6 (−0.4; 1.7)	0.9±5.0	−0.2±5.2	1.0 (−0.2; 2.2)
Fibre (g per 1000 kcal)	1.2±2.5^c^	0.5±2.1^c^	0.6 (0.1; 1.1)	1.2±2.7^c^	0.3±2.0	0.9 (0.4; 1.5)
Alcohol (en%)	−0.6±2.8^c^	−0.3±2.7	−0.2 (−0.8; 0.4)	−0.4±2.7^c^	−0.2±3.4	−0.1 (−0.8; 0.6)
Fruit intake incl. fruit juices (g per day)	20.2±116.8^c^	−28.5±126.6^c^	38.6 (14.3; 62.9)	7.4±125.4	−23.8±141.6	23.6 (−4.0; 51.3)
Vegetable intake (g per day)	6.1±83.0	2.6±78.7	8.4 (−8.6; 25.4)	15.7±91.9^c^	2.2±80.9	19.3 (1.0; 37.6)
Fibre intake from total bread intake (%)	0.4±1.5^c^	−0.0±1.1	0.3 (0.0; 0.5)	0.4±1.6^c^	0.1±0.9	0.3 (0.0; 0.6)
Fat intake from total bread spread intake (%)	−3.3±6.2^c^	−0.5±5.6	−2.1 (−3.3; −0.9)	−3.9±6.8^c^	−0.5±6.5	−2.6 (−4.1; −1.1)
Snack intake (kcal per day)	−59.6±146.0^c^	−72.3±222.0^c^	−15.0 (−44.5; 14.6)	−45.4±175.6^c^	−93.1±239.8^c^	14.8 (−22.0; 51.7)
Soft drink intake (kcal per day)	−15.6±66.9^c^	−13.7±55.4^c^	2.2 (−9.1; 13.6)	−21.9±57.4^c^	−10.6±52.3^c^	−8.0 (−18.5; 2.6)
High-fat dairy intake (g per day)	−18.0±55.6^c^	−7.6±51.1^c^	−11.6 (−21.7;−1.6)	−16.3± 52.1^c^	−13.7±46.8^c^	−6.5 (−16.1; 3.1)
Low-fat dairy intake (g per day)	20.8±157.0^c^	−12.9±158.7	33.7 (−0.3; 67.6)	12.5±182.1	−2.3±127.4	16.3 (−22.7; 55.2)
Dutch Healthy Diet index (0–80 scale)^d^	3.6±7.9^c^	0.3±7.7	3.4 (1.7; 5.0)	3.8±7.8^c^	0.3±7.8	3.8 (2.1; 5.5)
						
*Physical activity (PA)*						
Total PA (min per week)	152.5±1346.5	−80.1±1225.3	229.9 (−47.3; 507.1)	159.2±1552.6	98.8±1094.6	55.0 (−258.5; 368.6)
Light PA (min per week)	89.7±970.4	26.4±997.0	70.4 (−139.9; 280.8)	33.7±1270.8	56.7±875.2	−21.5 (−267.9; 225.0)
Moderate PA (min per week)	−2.9±649.6	−26.3±531.0	39.7 (−81.8; 161.2)	17.9±684.4	28.2±609.1	0.8 (−149.3; 150.8)
Vigorous PA (min per week)	65.7±415.7^c^	−80.2±412.7^c^	118.3 (33.8; 202.8)	107.6±429.7^c^	13.9±317.3	82.0 (−7.9; 171.9)
Physical fitness (m)	25.1±38.7^c^	2.3±37.4	22.6 (13.5; 31.7)	32.8±47.2^c^	10.0±40.8^c^	22.3 (10.7; 33.9)

Abbreviations: CI, confidence interval; CON, Control group; INT, Intervention group; PA, physical activity.

a Data are mean±s.d. or *β* (95% CI).

b From baseline to 12 months: dietary intake: INT *n*=138, CON *n*=134; physical activity (min/week): INT *n*=139, CON *n*=133; physical fitness: INT *n*=137; CON *n*=130; From baseline to 18 months: dietary intake: INT *n*=116, CON *n*=122; physical activity (min per week): INT *n*=117, CON *n*=122; physical fitness: INT *n*=105, CON *n*=118.

c Significant difference within group (*P*<0.05).

d From baseline to 12 months: INT *n*=138, CON *n*=133.

**Table 4:** Changes in quality of life from baseline to 12 months (*n*=271) and 18 months (*n*=233)^a^

	*From baseline to 12 months*			*From baseline to 18 months*		
	*INT*	*CON*	β *(95% CI)*	*INT*	*CON*	β *(95% CI)*
*n*	138	133		115	118	
Health transition	15.8±26.5^b^	0.4±23.2	14.5 (10.0; 18.9)	12.2±25.5^b^	−0.2±21.8	10.9 (5.9; 16.0)
General health	5.5±15.3^b^	3.0±14.6^b^	2.7 (−0.3; 5.7)	7.0±15.6^b^	2.4±15.8	4.4 (0.9; 7.9)
Physical functioning	3.4±13.1^b^	0.2±12.1	3.1 (0.2; 5.9)	2.7±14.4^b^	−1.0±14.0	3.4 (−0.1; 6.8)
Role physical	4.7±36.1	−0.4±35.8	4.3 (−3.1; 11.8)	3.5±40.3	2.1±36.5	0.3 (−7.9; 8.5)
Role emotional	4.8±34.5	3.1±29.9	−0.5 (−6.8; 5.8)	9.0±37.3^b^	−1.6±34.6	8.1 (0.6; 15.5)
Social functioning	2.2±20.3	−1.0±17.1	2.5 (−1.4; 6.5)	2.5±18.8	−2.3±19.0	4.5 (0.3; 8.7)
Bodily pain	2.6±21.2	−0.1±19.7	3.7 (−0.7; 8.0)	1.2±21.8	0.5±18.6	1.3 (−3.4; 6.0)
Vitality	4.5±12.3^b^	2.1±12.6	2.7 (−0.1; 5.5)	3.7±11.4^b^	1.2±14.5	3.1 (−0.2; 6.4)
Mental health	2.8±10.0^b^	−0.6±12.1	3.4 (1.0; 5.7)	3.4±11.8^b^	0.9±12.4	2.7 (−0.2; 5.6)
Physical component score	1.5±7.4^b^	0.3±6.2	1.4 (−0.1; 2.9)	0.9±7.7^b^	0.4±7.1	0.5 (−1.2; 2.3)
Mental component score	1.6±7.6^b^	0.4±6.9	1.0 (−0.5; 2.5)	2.4±8.3^b^	−0.1±8.7	2.5 (0.6; 4.5)

Abbreviations: CI, confidence interval; CON, Control group; INT, intervention group.

a Data are mean±s.d. or *β* (95% CI).

b Significant difference within group (*P*<0.05).
