# Supplementary material for: Effect and maintenance of the SLIMMER diabetes prevention lifestyle intervention in Dutch primary healthcare: a randomised controlled trial
Source: Nutr Diabetes. 2017 May 8;7(5):e268–. doi: 10.1038/nutd.2017.21 (PMC5518803; doi:10.1038/nutd.2017.21)
Supplement: Supplementary Information [file nutd201721x1.docx]

Online-Only Supplemental Material.

T1

Post

**Measurements**

- OGTT
- Physical examination
- Questionnaires

**Intervention group**

*Core programme*

- 10-month dietary and physical activity intervention

*Maintenance programme*

- Sports clinics
- Concluding meetings

T0

Pre

**Measurements**

- Oral Glucose Tolerance Test (OGTT)
- Physical examination
- Questionnaires

**Randomisation**

**Control group**

- Usual care
- Written information on healthy lifestyle

T2

6 months post

**Measurements**

- OGTT
- Physical examination
- Questionnaires

*Maintenance programme*

- Return visit

Supplementary Figure S1. Timeline of the SLIMMER intervention.

Supplementary Table S1. Changes in clinical and metabolic characteristics from baseline to 12 months (*n*=240) by manner of recruitment^a^

|  | Laboratory glucose test (IFG) | | | Diabetes Risk Test (risk score) | | |  |
| --- | --- | --- | --- | --- | --- | --- | --- |
|  | INT | CON | β (95%CI)^b^ | INT | CON | β (95%CI)^b^ | *p*-value for interaction |
| *n* | 64 | 66 |  | 57 | 53 |  |  |
| Weight (kg) | -3.5 ± 4.7^*^ | -0.5 ± 2.5 | -2.9 (-4.2; -1.6) | -3.0 ± 4.9^*^ | 0.5 ± 4.2 | -3.3 (-4.9; -1.6) | 0.612 |
| BMI (kg/m^2^) | -1.2 ± 1.6^*^ | -0.2 ± 0.9 | -1.0 (-1.4; -0.6) | -1.0 ± 1.6^*^ | 0.2 ± 1.4 | -1.1 (-1.6; -0.5) | 0.764 |
| Waist circumference (cm) | -5.7 ± 5.3^*^ | -1.3 ± 3.6^*^ | -4.3 (-5.9; -2.8) | -5.2 ± 5.8^*^ | -0.9 ± 5.5 | -3.8 (-5.8; -1.7) | 0.775 |
|  |  |  |  |  |  |  |  |
| Fasting glucose (mmol/l) | -0.2 ± 0.7^*^ | 0.1 ± 0.7 | -0.4 (-0.6; -0.1) | -0.1 ± 0.6 | -0.1 ± 0.6 | -0.0 (-0.2; 0.2) | **0.016** |
| 2-h glucose (mmol/l)^c^ | -0.5 ± 2.2 | 0.4 ± 3.2 | -1.2 (-2.1; -0.3) | -0.4 ± 2.5 | -0.0 ± 1.6 | -0.5 (-1.1; 0.1) | 0.613 |
| Fasting insulin (pmol/l) | -16.4 ± 38.8^*^ | 3.1 ± 33.2 | -14.2 (-25.6; -2.8) | -10.2 ± 37.3 | -0.4 ± 46.4 | -14.1 (-26.7; -1.5) | 0.746 |
| HOMA-IR | -0.38 ± 0.89^*^ | 0.08 ± 0.78 | -0.35 (-0.61; -0.08) | -0.23 ± 0.85 | -0.01 ± 1.02 | -0.32 (-0.60; -0.04) | 0.862 |
| HbA1c (% (mmol/mol)) | -0.19 ± 0.20^*^  (-2.06 ± 2.16^*^) | -0.07 ± 0.28^*^  (-0.73 ± 3.07^*^) | -0.12 (-0.21; -0.04)  (-1.35 (-2.29; -0.41)) | -0.12 ± 0.21^*^  (-1.28 ± 2.25^*^) | -0.07 ± 0.15^*^  (-0.74 ± 1.64^*^) | -0.04 (-0.11; 0.02)  (-0.48 (-1.19; 0.23)) | 0.138  0.138 |
| Total cholesterol (mmol/l) | -0.12 ± 0.81 | -0.06 ± 0.94 | -0.04 (-0.30; 0.21) | -0.18 ± 0.79 | -0.05 ± 1.01 | -0.09 (-0.36; 0.19) | 0.834 |
| HDL cholesterol (mmol/l) | 0.04 ± 0.16 | 0.03 ± 0.13 | 0.02 (-0.03; 0.07) | -0.00 ± 0.20 | 0.00 ± 0.16 | -0.01 (-0.07; 0.05) | 0.461 |
| LDL cholesterol (mmol/l) | -0.15 ± 0.77 | -0.15 ± 0.89 | -0.00 (-0.24; 0.23) | -0.05 ± 0.75 | -0.06 ± 0.87 | 0.01 (-0.23; 0.25) | 0.898 |
| Triglycerides (mmol/l) | -0.07 ± 0.56 | 0.10 ± 0.80 | -0.18 (-0.41; 0.06) | -0.20 ± 0.71 | 0.01 ± 0.72 | -0.12 (-0.34; 0.09) | 0.932 |
| Systolic blood pressure (mmHg)^c^ | -2.9 ± 12.0 | -0.7 ± 12.2 | -1.6 (-5.7; 2.4) | -2.0 ± 11.0 | -3.1 ± 10.4^*^ | 1.8 (-1.8; 5.4) | 0.224 |
| Diastolic blood pressure (mmHg)^c^ | -4.3 ± 7.9^*^ | -2.4 ± 7.1^*^ | -0.6 (-3.1; 1.8) | -3.3 ± 7.1^*^ | -2.3 ± 7.1^*^ | -1.0 (-3.6; 1.5) | 0.856 |

Boldface indicates statistical significance (*p* < 0.05).

^a^ Data are mean ± SD or β (95% CI).

^b^ β (95% CI) for fasting glucose, 2-h glucose, fasting insulin, HOMA-IR, HbA1c, total cholesterol, HDL cholesterol, LDL cholesterol, triglycerides, systolic and diastolic blood pressure were adjusted for medication use.

^c^ Laboratory glucose test: 2-h glucose: INT *n*=61, CON *n*=62; systolic and diastolic blood pressure: INT *n*=62, CON *n*=62; Diabetes Risk Test: 2-h glucose: INT *n*=56, CON *n*=53; systolic and diastolic blood pressure: INT *n*=56, CON *n*=53.

^*^ Significant difference within group (*p*<0.05).

BMI, Body Mass Index; CON, Control group; HbA1c, glycated haemoglobin; HDL, high-density lipoprotein; HOMA-IR, homeostasis model assessment insulin resistance; IFG, impaired fasting glucose; INT, Intervention group; LDL, low-density lipoprotein; PA, physical activity.
